# Preparation of Biomass Biochar with Components of Similar Proportions and Its Methylene Blue Adsorption

**DOI:** 10.3390/molecules28176261

**Published:** 2023-08-26

**Authors:** Min Hou, Yudan He, Xuewen Yang, Yuchun Yang, Xu Lin, Yongxing Feng, Huan Kan, Huirong Hu, Xiahong He, Can Liu

**Affiliations:** 1 National Joint Engineering Research Center for Highly-Efficient Utilization Technology of Forestry Resources, Southwest Forestry University, Kunming 650224, China; houmin124@163.com (M.H.); yudan4560623@163.com (Y.H.); 13351392986@163.com (X.Y.); ynlcyyc87038551@163.com (Y.Y.); linxu@swfu.edu.cn (X.L.); 18087209470@163.com (Y.F.); 13700650213@163.com (H.K.); hhrxl@163.com (H.H.); 2 Yunnan Academy of Forestry and Grassland, Kunming 650201, China; 3 Lincang Academy of Forestry Sciences, Lincang 677000, China

**Keywords:** biomass, physicochemical properties, adsorption properties, methylene blue

## Abstract

Rapeseed straw, bagasse, and walnut peel have a large amount of resource reserves, but there are few technologies for high value-added utilization. In the research of biochar, walnut green husk is rarely used as raw material. In addition, the three main components of biomass (lignin, cellulose, and hemicellulose) are present in similar proportions, and the differences between the physical and chemical properties of biochar prepared with similar amounts of biomass raw materials are not clear. Using three kinds of biomass of the same quality as raw materials, biochar was prepared via pyrolysis at 400 °C, and activated carbon was prepared via CO_2_ activation at 800 °C. The results showed that the pore numbers of the three kinds of biochar increased after activation, resulting in the increase of the specific surface area. The resulting numbers were 352.99 m^2^/g for sugarcane bagasse biochar (SBB)-CO_2_, 215.04 m^2^/g for rapeseed straw biochar (RSB)-CO_2_, and 15.53 m^2^/g for walnut green husk biochar (WGB)-CO_2_. Ash increased the amount of carbon formation, but a large amount of ash caused biochar to form a perforated structure and decreased the specific surface area (e.g., WGB), which affected adsorption ability. When the three main components were present in similar proportions, a high content of cellulose and lignin was beneficial to the preparation of biochar. The adsorption value of MB by biochar decreased with the increase of biomass ash content. After activation, the maximum adsorption value of MB for bagasse biochar was 178.17 mg/g, rapeseed straw biochar was 119.25 mg/g, and walnut peel biochar was 85.92 mg/g when the concentration of methene blue solution was 300 mg/L and the biochar input was 0.1 g/100 mL at room temperature. The adsorption of MB by biochar in solution occurs simultaneously with physical adsorption and chemical adsorption, with chemical adsorption being dominant. The optimal MB adsorption by SBB-CO_2_ was dominated by multimolecular-layer adsorption. This experiment provides a theoretical basis for the preparation of biochar and research on its applications in the future.

## 1. Introduction

Due to industrial production and human activities, the pollution of water resources is becoming increasingly serious, especially the pollution caused by dye wastewater [1,2,3,4]. At present, the treatment methods for hazardous dyes can be divided into chemical, biological, physical, and some optimized combination processes [5]. Among previous research methodologies, adsorption is the most ideal method that has been studied in recent years [6,7]. The activated carbon adsorption method has successfully been applied to the treatment of dye wastewater using physicochemical techniques, and its main features are simple operation, high efficiency, and ease of implementation. There are various types of activated carbon, among which biochar is easy to obtain, low in cost, and recyclable. Numerous scholars have studied the pyrolysis carbon of agroforestry biomass [8,9,10,11] and found that even when the same activation process is used, there are considerable differences in the performances of various biochars, and the performance differences become increasingly obvious with greater differences in the structure and composition of the biomass. The surface properties and structure are closely related to the adsorption performance. Walnut (*Juglans regia* L.) [12], sugarcane (*Saccharum officinarum* L.) [10], rape (*Brassica campestris* L.) [13], and other agroforestry products are all high-yield crops in the world, and the comprehensive utilization of waste generated from their processing has become an inevitable issue in the food industry. Traditional waste is mainly discarded in piles and burned, which not only causes considerable energy waste but also causes serious environmental pollution if not treated quickly. Walnut green husk is a thick layer of green peel on the outside of walnuts that contains a variety of secondary metabolic substances, which can affect the ecological balance and cause serious water pollution at walnut processing sites; every year, the weight of green husks produced is four to five times the weight of dried walnuts [12]. Slow processing of this material causes serious environmental pollution [14]. Rapeseed straw and sugarcane bagasse [15] are the bulk byproducts of modern agricultural products and potentially serve as large stocks of raw material for biochar. Walnut green husk, sugarcane bagasse, and rapeseed straw are mainly composed of cellulose, hemicellulose, and lignin, which are abundant in carbon and are potentially sufficient resources for the preparation of biomass charcoal [16].

Overall, the preparation of biochar adsorbents is an important method for the clean utilization of biomass and obtaining renewable energy. Physical activation, chemical activation, heat treatment, and impregnation are the most common methods for activated carbon modification [17]. Among them, chemical activation and modification is an important method to control the surface functional groups and chemical properties of activated carbon. The industrialization of physical activation method is the largest and more green [18]. Moreover, one-step (co-pyrolysis of biomass and activation) and two-step (carbonization and activation, respectively) activation processes also have been reported [9,19,20]. In comparison, the two-step process is conducive to the high yield of biochar and further gasification of biochar with relatively low porosity, to form a large number of new pores, and it had obtained more recognition [21]. Some studies on biochar prepared from sugarcane bagasse and rapeseed straw have been reported, but there are fewer reports related to the preparation of activated carbon from walnut green husk [22]. Moreover, due to the different compositions of the three biomasses, the pattern of influence of the composition on the physical and chemical properties of biochar prepared using the same process is not clear. The intrinsic connection between the adsorption performance of biochar prepared via the same process and the composition of the biomass is also unclear. A comparative analysis of the structural properties and adsorption mechanisms of different biomasses with similar proportions is also lacking.

**Figure 1:**
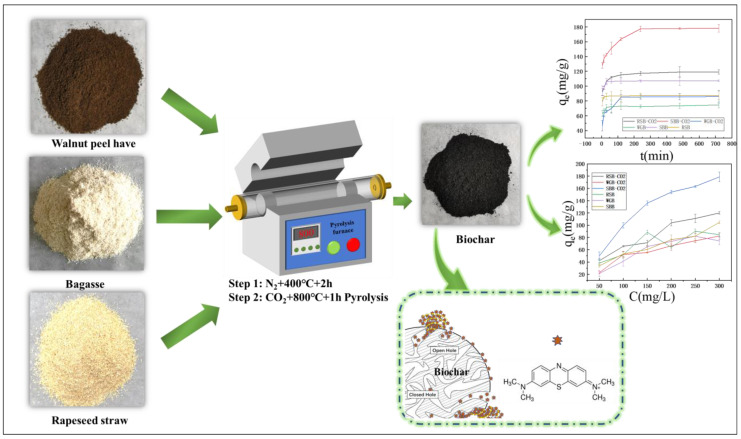
Flow chart of the preparation route.

Considering the above issues, we took three types of biomasses with similar components (walnut peel, rapeseed straw, and sugarcane bagasse) as raw materials and used CO_2_ gas to prepare three types of biochar through physical activation methods. The relationship between adsorption properties of biochar prepared using the same process and biomass composition is discussed (Figure 1). We tested three biochar samples with similar fractions but different sources and used BET, SEM, FTIR, XRD, and XPS for structural characterization. Adsorption experiments were conducted using the prepared biochar, and the adsorption ability of biochar on the pigment model compound methylene blue (MB) was investigated. Furthermore, the differences in adsorption performance of three biochar samples with similar components were studied, providing a theoretical basis for the preparation and application of biochar. In conclusion, the study of adsorbents prepared from walnut green bark, sugarcane bagasse, and rapeseed straw biomass are of great significance for the comprehensive utilization of such biomass, which has excellent potential for energy development.

## 2. Results and Discussion

### 2.1. Analysis of Biomass Composition

The main components of the three biomasses are lignin, cellulose, and hemicellulose, and the lignin/cellulose/hemicellulose ratios of the three biomasses were determined to be 1:1.9:0.7 for green husk, 1:2.2:1.2 for sugarcane bagasse, and 1:2.0:0.8 for rapeseed straw. The proportions of the three major components of the biomasses were similar but had certain differences, as shown in Table 1. The cellulose content and hemicellulose content of sugarcane bagasse were relatively high, and the analysis showed that the physiological form of bagasse had a high content of cellulose as well as a regular structure; sugarcane is the main source crop of sugar, so its hemicellulose content was also found to be relatively high. Walnut shell green bark has a high ash content. The differences among the three major components of biomass had different effects on biochar preparation.

When the carbonization temperature was 400 °C, the yields of walnut husk, sugarcane bagasse, and rapeseed straw were 38.80%, 31.50%, and 35.00%, respectively; the final yields were 30.33%, 23.81%, and 24.26% for the three biomasses after activation with CO_2_. The decrease in carbon yield was similar for the three biomasses after activation. The higher yield of walnut green husk was attributed to its higher ash content (11.30%), as shown in Table 1.

### 2.2. Pore Structure Analysis

**Figure 2:**
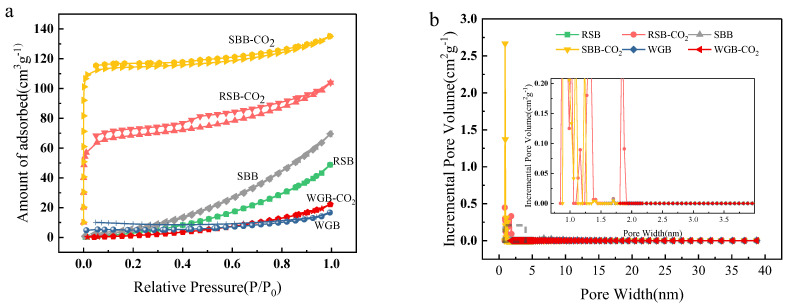
Isotherms (**a**) and pore size distributions (**b**) of N_2_ adsorption analysis of different walnut green bark, sugarcane bagasse, and rapeseed straw biochars.

As shown in Figure 2a, the N_2_ adsorption–desorption isotherms of sugarcane bagasse biochar (SBB)-CO_2_ and rapeseed straw biochar (RSB)-CO_2_ both exhibited type IV adsorption isotherms with an H4 hysteresis ring, demonstrating micro-mesoporous adsorption characteristics. The type IV adsorption isotherm rose rapidly at lower relative pressures, and the curve was convex. At higher P/P0 values, the adsorption increased slightly with relative pressure. As the saturation pressure reached the maximum, there was a certain hysteresis back to the ring, corresponding to an adsorption system in which capillary coalescence of the porous adsorbent occurs. After the mesoporous capillary coalescence was filled, the adsorption isotherm continued to rise, indicating that the biochar still possessed large pores, resulting in multilayer adsorption. The H4-type hysteresis ring indicates that this activated carbon was characterized by a mixture of micropores and mesopores, with narrow fissure pores [23]. Type II isotherm curves were observed for the other four biomasses, and the analysis shows that these types of biochar had a strong adsorption effect on the surface with adsorbents, which is characteristic of typical macroporous structures. From the pore size distribution diagram in Figure 2b, it can be seen that the microporous structure of activated carbon was more developed after CO_2_ activation, which also caused sugarcane biochar and RSB to have abundant narrow and uneven pores, indicating that CO_2_ activation was beneficial for the generation of activated carbon microporosity.

As seen in Table 2, the specific surface area of the three biochars under the carbonization conditions at 400 °C was the largest for sugarcane bagasse at 24.86 m^2^/g, followed by rapeseed straw at 15.63 m^2^/g, and walnut green husk at 6.57 m^2^/g. The microporous volume of the three biochars accounted for approximately 20%, with the average pore size ranging from 5.52 to 7.86 nm, with mesopores as the main pore type. On the one hand, this was due to the high content of the volatile fraction in the biomass material during the carbonization process, and the high concentration of the volatile fraction cleared some of the filling obstructions, such as tar, from the structure, resulting in the conversion of microporous linkages into mesopores; that is, the pore size increased. On the other hand, the increased degree of pyrolysis caused the pore wall of the biochar to collapse and the pore structure of the biochar to deform, reorganize, and close. The specific surface area and micropore volume of activated carbon increased substantially after CO_2_ activation. The specific surface area of SBB-CO_2_ was the largest at 352.99 m^2^/g, followed by RSB-CO_2_ at 215.04 m^2^/g, and then walnut green husk biochar (WGB)-CO_2_ at only 15.53 m^2^/g. The specific surface area of the activated biochar was more than two and up to fifteen times higher than before activation. The percentage of micropores was substantially increased, with that for the SBB increasing by 62%. This was because CO_2_ entered the interior of the activated carbon, which expanded the pores of the activated carbon. Since the molecular diameter of CO_2_ is small, the activated carbon prepared via the CO_2_ activation method was mainly microporous. However, the effect on the biochar of walnut exocarp was very weak, and it was speculated that the large amount of ash affected the pore formation, resulting in the formation of mostly macroporous structures, which affected the adsorption performance. Moreover, the lower lignin content of walnut shell exocarp and the low cellulose content affected the carbon formation of biomass.

### 2.3. SEM Analysis of Apparent Morphology

**Figure 3:**
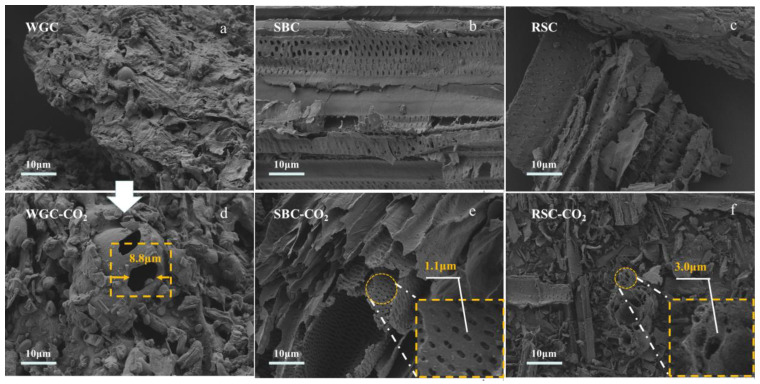
SEM images of the surface structures of activated carbon. (**a**–**c**) Micromorphology of biochar, (**d**–**f**) Micromorphology of biochar after CO_2_ activation.

We observed the surface morphology of the biochar. As shown in Figure 3, obvious porous structures were observed in the SEM images of all three biochars, and WGB showed a granular porous structure, while SBB and RSB showed tubular porous structures. The interior of the WEB presented a layered structure and formed large pores, with a maximum pore size of 8.8 µm. This unique structure of WGB may ultimately cause its relatively low specific surface area. Both SBB and RSB maintained their unique fibrous structures before and after activation, and both biochars had a high number of pores, which were mainly arranged in strips with a relatively regular arrangement. The biochar after CO_2_ activation appeared to have honeycomb-like cavities and formed a denser pore structure. Small pore-like structures were clearly observed on the surface of rapeseed straw, which was significant for the adsorption process.

The surface layered structure of the biochar samples increased after CO_2_ activation, and the carbon skeleton was stretched and had many irregular pores with increased surface pore density, indicating that CO_2_ etched and expanded the carbon layer of the biochar. This effect decreased the pore size and increased the specific surface area; while a small number of macropores were observed on the surface of WGB-CO_2_, the skeleton was stretched severely and collapsed, and the degree of breakage was relatively high. In addition to the structural composition of walnut green husk (Table 1), the analysis showed that the three biomasses had similar proportions of cellulose, lignin, and hemicellulose, but the relative contents of cellulose and lignin were lower in walnut green husk than in the other two biomasses. Lignin and cellulose are important factors affecting the micromorphology of biochar. Although the composition ratios of cellulose, lignin, and hemicellulose of the biomasses were similar, the ash content of the three kinds of biochar had the greatest influence. Moreover, because there is less carbon skeleton formation in walnut green husk at high temperature, the volatile fraction content was significantly higher than that of the other two biomasses, which resulted in an uneven macropore structure and thus a lower specific surface area.

### 2.4. XRD Analysis of Biochar

**Figure 4:**
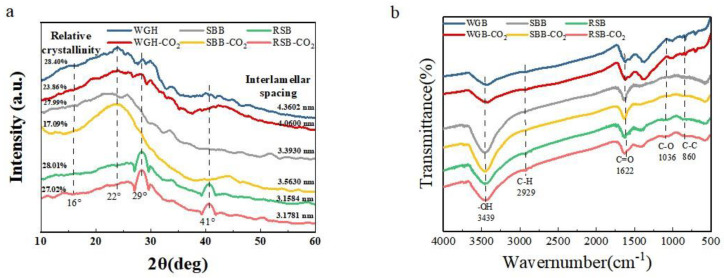
XRD (**a**) and FTIR (**b**) spectra of three kinds of biomass before and after activation.

The crystalline structures of the materials are depicted in Figure 4a. The degree of cellulose polymerization in the three prepared biocarbon systems decreased rapidly after baking at 400 °C, and by comparison, after CO_2_ activation to form active cellulose followed by cleavage of the glycosidic bonds, the system had an amorphous structure, and the crystallinity decreased significantly; this was due to side chain removal and dehydration of the cellulose caused by baking. A portion of the hydrogen bonds were broken, and the macromolecular chains were cleaved, thus destroying the crystalline structure to a certain extent and excessively degrading it. The diffractograms of the activated carbons are very similar, with extended signals between 18 and 30° and 40 and 46°, indicating the presence of amorphous carbon.

In the spectrum of WGB, the diffraction peaks at approximately 16° and 22° disappeared, and the crystallinity decreased. These are the characteristic absorption peaks of two typical type I lignocelluloses: the former corresponds to the noncrystalline area of cellulose, and the latter is the 101/002 diffraction absorption peak of the crystalline area of cellulose. Some of the crystalline areas in walnut green husk were converted into noncrystalline areas, which increased the number of adsorption sites and helped improve its adsorption capacity [24]. Diffraction peaks appeared near 2θ = 22.1°, 26.1°, 50°, etc., in the spectrum of SBB, corresponding to oxides of elemental Si in the biochar; diffractograms showing diffraction lines indicating the presence of silica (quartz), alumina, calcium oxide, magnesium oxide, iron trioxide, and potassium oxide [25,26], which disappear after CO_2_ activation, have been reported. The spectrum of RSB had strong diffraction peaks at 2θ = 29° and 2θ = 41°, with the former corresponding to Si elemental oxide and the latter corresponding to the 100-crystal surface of a graphite structure, showing signs of graphite microcrystals, which is typical of amorphous carbon materials with low graphitization, indicating that the structure of lignocellulose in the carbon matrix was destroyed in the biochar [27].

The composition of the groups on the surface of the material is depicted in Figure 4b. The peak near 3444 cm^−1^ was attributed to the characteristic O–H or N–H stretching vibration of hydroxyl or carboxyl groups [28]. The peak at 2929 cm^−1^ was attributed to the CH stretching vibration of –CH_3_– or –CH_2_– [29]. The peak at 1615 cm^−1^ was attributed to the characteristic C=C/C=N/C=O stretching vibration of the activated carbon surface [30]. The peak at 1036 cm^−1^ was attributed to the C–O–C and C–O stretching vibrations or O–H bending vibrations in alcohols, phenols, and carboxyl groups [31]. The peak at 836 cm^−1^ was the characteristic peak of the benzene ring (C=C) generated during the activation process [32]. The graphical analysis showed that different biomasses gave rise to different peaks at 2929 cm^−1^ and 1078 cm^−1^. The peak at 2926 cm^−1^ disappeared, indicating that the high temperature destroyed the CH functional groups on the surface of the biomass material and that the removed mixture also contained aromatic rings, O–H, C–O, and other bonds. Significant peaks at 836 cm^−1^ were observed for WGB and WGB-CO_2_, indicating that high-temperature char activation is favorable for the generation of graphite and aromatized structures on the surface of activated carbon. In summary, all six biochars were dominated by oxygen-containing functional groups and aromatic ring skeletal structures, which provided certain adsorption active sites for the adsorption of MB and promoted the adsorption of several biomass activated carbons.

### 2.5. XPS Analysis of Biochar

**Figure 5:**
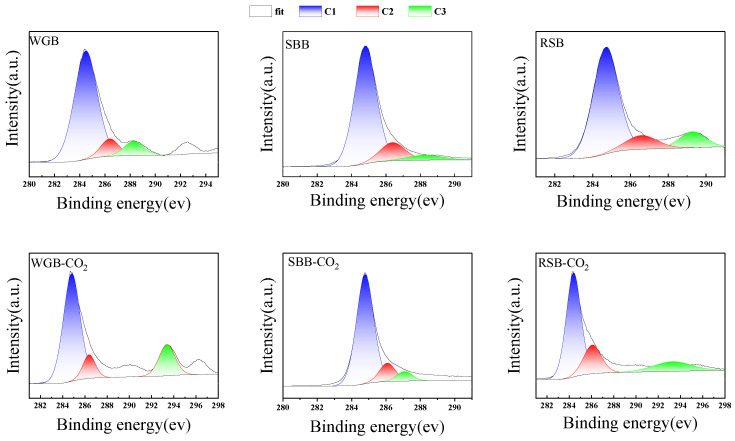
XPS survey spectra of different biochars.

To more thoroughly analyze the elemental distribution on the surface of the biochar, XPS analysis of the material was performed. In Figure 5 and Table 3, it can be seen that the carbon content of SBB was the highest before and after carbonization, while the oxygen content of RSB was the highest. After activation, the oxygen content of biochar decreased, with that of SBB decreasing the most. The analysis showed that the dispersion structure with multiple fibers was beneficial to the deoxygenation of biomass. By combining these results with the XRD data analysis, it was seen that a higher carbon content resulted in the formation of more graphite-like structures. The combination of C and O on the surfaces of WGB, SBB, and RSB changed after activation treatment, with C–C/C=C (C1) and C-O (C2) combinations dominating. The C–C bond content of activated WGB and RSB decreased, the noncarbohydrate content decreased, and the C–C bond content of activated SBB increased slightly. The increased proportion of activated biochars C2 and C3 indicates that more C=O and O–C=O groups are formed on the carbon surface, and the increase of such groups is conducive to the generation of followed chemisorption [27]. The SBB-CO_2_ biochar formed more C=C/C–C (C1) on the surface, with a relative content as high as 80.65%, while the WGB-CO_2_ biochar only had 69.44%, and the RSB-CO_2_ biochar only had 63.51%. The analysis shows that the biochar prepared using the sample with high cellulose content in the biomass composition can form a more graphitized structure. The surface of SBB-CO_2_ biochar has more C–C/C=C relative content; that is, more graphite and aromatic structures are formed on the surface of activated carbon, which can generate a π–π interaction with the thick ring structure of MB, which is conducive to adsorption [30]. The increased proportion of bagasse biochar is higher than the other two, and the adsorption capacity should be stronger in theory.

### 2.6. Effects of Adsorption Process Conditions on the Adsorption Properties of Naphthalene

**Figure 6:**
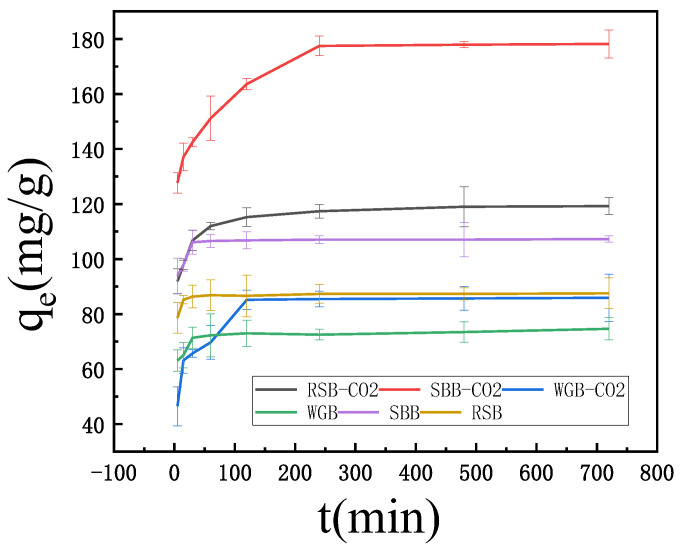
Effect of adsorption time on the adsorption efficiency of different adsorbents.

The results displayed in Figure 6 and Table 4 show the effect of each biochar on the adsorption of MB at different adsorption times, with an increase in adsorption as the adsorption time increased. During the first 30 min, the amount of MB adsorbed increased significantly, and the adsorption rate increased. With increasing adsorption time, the amount of MB adsorbed increased only slightly. This was because both the surface functional groups of the baking carbon and the concentration of MB in the solution were maximum in the initial stage, the mass transfer driving capacity was the highest, and the rate of adsorption was the highest [30]. At an adsorption time of 30–240 min, the increase in adsorption amount leveled off because the concentration of MB gradually decreased during the adsorption process, the driving force decreased, and the rate of adsorption decreased. When the adsorption time exceeded 240 min, adsorption no longer occurred. The changes in adsorption tended toward dynamic equilibrium, so 240 min was chosen as the reaction condition. The figure shows that the MB adsorption of all three biochar samples increased after the activation of CO_2_. The order of MB adsorption of the six biochars was as follows: SBB-CO_2_ > RSB-CO_2_ > SBB > RSB > WGB-CO_2_ > WGB. The MB adsorption of SBB-CO_2_ was the largest, with a value of 178.17 mg/g. The SBB-CO_2_ sample was 84% microporous and 16% mesoporous or macroporous, which provided a “Macroporous–Mesoporous–Microporous” transport channel for MB adsorption and diffusion. At the same time, it had dense graphitization structure, aromatization structure, and surface oxygen-containing functional groups; the existence of these characteristics could allow MB to easily enter the adsorption site and carry out physical and chemical adsorption. Even if the specific surface area was low, it still had the highest adsorption capacity (178.17 mg/g). Therefore, the MB adsorption mechanism of SBB-CO_2_ biochar was dominant via the pore diffusion mechanism and π–π interaction.

**Figure 7:**
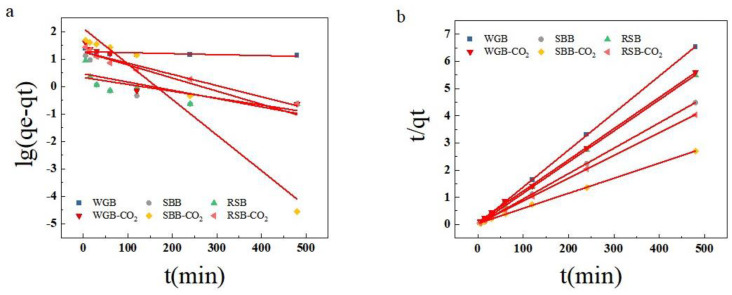
Fitting curve of biochar adsorption kinetics of different raw materials: quasi-first-order kinetic model (**a**) and quasi-second-order kinetic model (**b**).

Figure 7 and Table 5 show that the fitting correlations of the pseudo-second-order kinetic equations for the biochar samples were all greater than those of the pseudo-first-order kinetic equations. In the pseudo-second-order kinetic equations, the correlation between the adsorption of naphthalene by biochar was R^2^ > 0.999. At this point, the theoretical adsorption value q_e_ was close to the experimental adsorption amount value q_exp_. Therefore, the solution adsorption of MB by activated samples was consistent with the pseudo-second-order kinetic equation. The results indicated that biochar can adsorb naphthalene in two ways, physical and chemical, with chemisorption being the dominant mechanism.

**Figure 8:**
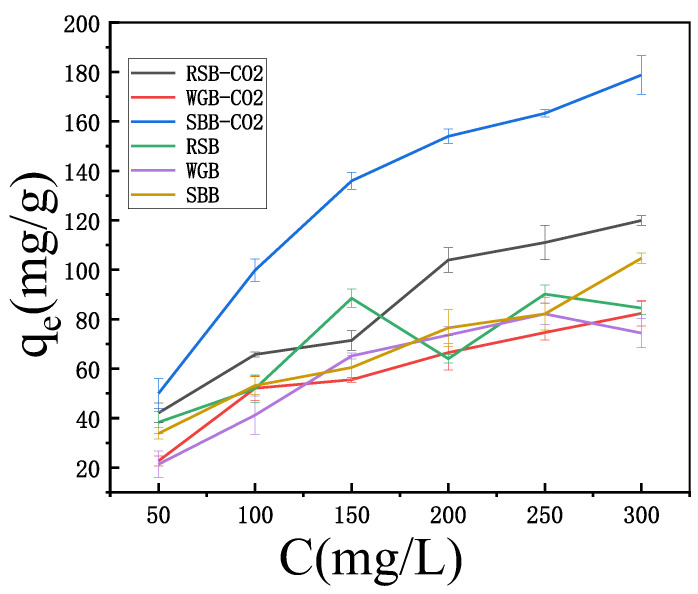
Effect of initial concentration on the adsorption effect.

The adsorption capacities of the six carbon materials with increasing initial mass concentrations of MB from 50 mg/L to 300 mg/L are shown in Figure 8. In general, the adsorption capacity increased gradually with the increase in the initial MB mass concentration, which was due to the increase in the adsorbent concentration, the increase in the concentration difference between the adsorbent surface and the main body of the solution, the increase in the driving force of adsorption, and the increase in the adsorption capacity, and therefore the decrease in the removal rate under the other conditions.

**Figure 9:**
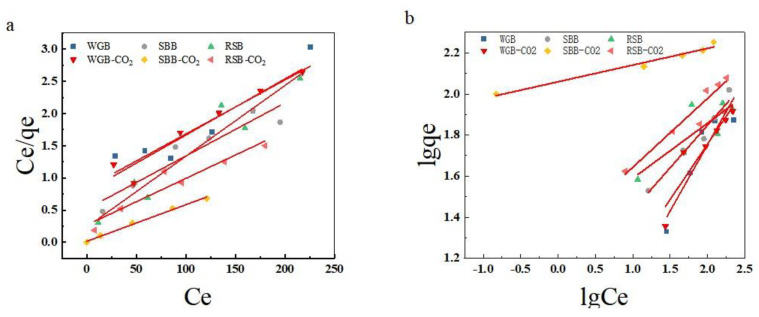
Isothermal fitting adsorption curve of different raw material biochars: Langmuir isothermal fitting curve (**a**) and Freundlich isothermal fitting curve (**b**).

Figure 9 and Table 6 illustrate the adsorption isotherm model parameters fitted analytically to the MB adsorption data of biochar by utilizing the Langmuir and Freundlich equations. The results of the analytical fitting of the MB adsorption data for the six biochars showed that the correlation of the fitted Freundlich equations for SBB-CO_2_ and RSB-CO_2_ biochars was higher than that of the Langmuir equation, indicating that the adsorption of MB by these two biochars was more consistent with the Freundlich model. In contrast, WGB-CO_2_ exhibited monolayer adsorption. From the fitting results for the Freundlich equation, it can be seen that with larger values of KF, the order of the adsorption strength of MB by biochar was as follows: SBB-CO_2_ > RSB > RSB-CO_2_ > SBB > WGB-CO_2_ > WGB. An n value less than 1 indicates an unfavorable adsorption process, 1 < n < 2 indicates a favorable adsorption process, and 2 > n indicates that the adsorption process of SBB-CO_2_ and RSB-CO_2_ is favorable, and adsorption occurs smoothly.

## 3. Discussion

According to these analyses, we prepared biochar from three biomasses (walnut green husk, sugarcane bagasse, and rapeseed straw) with similar ratios of cellulose, lignin, and hemicellulose using CO_2_ as the activating agent. We analyzed the biochars, and the specific surface area of the activated biochars increased substantially. The maximum specific surface area of the activated SBB was 352.99 m^2^/g. WGB-CO_2_ had a minimum specific surface area of 15.53 m^2^/g because the raw material contained more ash and less cellulose and formed larger voids. The biochar formed by rapeseed straw underwent some graphitization. The C–C/C=C, C–O, and C=O bonding ratios of the biochars increased after activation, and the increase was the largest for SBB. SBB and RSB showed good adsorption of MB, and the amount of activated carbon in the sugarcane bagasse reached 178.17 mg/g. The microporous structure and polar groups on the carbon surface were favorable for the adsorption of the pigment by the biochars. The adsorption of MB by the biochars conformed to the second-order kinetic equation, which describes a mixture of chemisorption and physisorption, with chemisorption dominating. The parameters of the adsorption isotherm model indicated that multimolecular-layer MB adsorption predominated. This study demonstrated the MB adsorption performance of biochar using the gas activation method and compared the differences in the structure and adsorption performance of biochars prepared from biomasses with similar proportions of three components, thus providing new suggestions for the efficient utilization of biomass.

## 4. Materials and Methods

### 4.1. Experimental Materials

Walnut green husk was provided by the Yangbi Walnut Institute of Yunnan Forestry and Grassland Academy of Sciences, sugarcane bagasse was purchased from Yunnan Kunming farmers’ market, and rapeseed straw was purchased from Luoping farmers’ market. The surface and internal residues were removed, dried at 105 °C for 48 h, crushed, and passed through a 100-mesh sieve and set aside in an airtight container for future use. AR reagents, such as methylene blue, concentrated sulfuric acid, phosphoric acid, ZnCl_2_, KOH, and ethanol, were provided by Shanghai Titan Technology Co., Ltd. (Shanghai, China). Unless otherwise stated, all reagents were used as is, and no further purification was needed. Deionized (DI) water was used throughout the study.

### 4.2. Preparation of Biochar

The biomass samples (10–30 g) were heated in a tube furnace to 400 °C at a rate of 10 °C/min under a N_2_ atmosphere (150 mL/min), maintained at the carbonization temperature for 2 h, and then cooled to room temperature. For physical activation, the carbonized biomass was heated to 800 °C at a rate of 10 °C/min under a CO_2_ atmosphere (150 mL/min) and maintained at the activation temperature for 1 h. We used water and ethanol to rinse the biochar to neutral and then dried it at 105 °C and placed it in a sealed container for use. The three biomasses, walnut green husk, sugarcane bagasse, and rapeseed straw, were named WGH, SB, and RS, respectively; the charcoals prepared at 400 °C were named WGB, SBB, and RSB; and the activated carbons prepared via activation of CO_2_ were named WGB-CO_2_, SBB-CO_2_, and RSB-CO_2_.

The yield of biochar is defined as the final mass of the product after carbonization, washing, and drying. The yield is determined using the following equation:

(1) $$\;\mathrm{yield}\left(\%\right)=\frac{W_{C}}{W_{m}}\times 100\%\;$$

where W_c_ and W_m_ are the final activated carbon dry weight (g) and precursor dry weight (g), respectively.

### 4.3. Methods

The biochar morphology was analyzed using an FEI Verios 460 scanning electron microscope (SEM). For the nitrogen adsorption–desorption (BET) analysis, the specific surface area and pore size were determined using a Micromeritics ASAP-2020 physisorber (Micromeritics, Norcros, GA, USA). The specific surface area was determined using the Brunauer‒Emmett–Teller method, the micropore volume and external surface area were determined using the t-plot method, and the mean pore size was determined using the BJH method. UV–visible spectra were obtained with a Shimadzu UV-2600 spectrometer (Shimadzu, Kyoto, Japan). Fourier transform infrared (FT-IR) spectra were obtained using the KBr pellet technique on a Nicolet iS5 spectrometer (Thermo Scientific, Waltham, MA, USA) at Thermal Sciences in the primary transmission mode, and 8 scans at a resolution of 1 cm^−1^ were accumulated to obtain one spectrum. X-ray photoelectron spectroscopy (XPS) was characterized primarily by using a K-Alpha spectrometer and a single X-ray source Al Kα excitation (1486.6 eV). Binding energy calibration for C1s was at 284.8 eV. A Rigaku D/max220 X-ray (Rigaku, Tokyo, Japan) diffractometer was used for analysis, and the scanning step was 0.02°. XRD test conditions include a Cu Ka ray source, tube voltage 40 kV, tube current 40 mA, 2θ angle range 5°~80°, and scanning rate 2 °/min. The relative contents of cellulose and lignin in the samples were determined using the NREL three-element standard; the average values of three parallel samples were determined for each sample, and an Agilent HPLC 1260 (Agilent, Palo Alto, CA, USA) was used.

### 4.4. Study of the Adsorption Performance of Activated Carbon

#### 4.4.1. Adsorption Experiments

(1) Standard curve plotting

**Figure 10:**
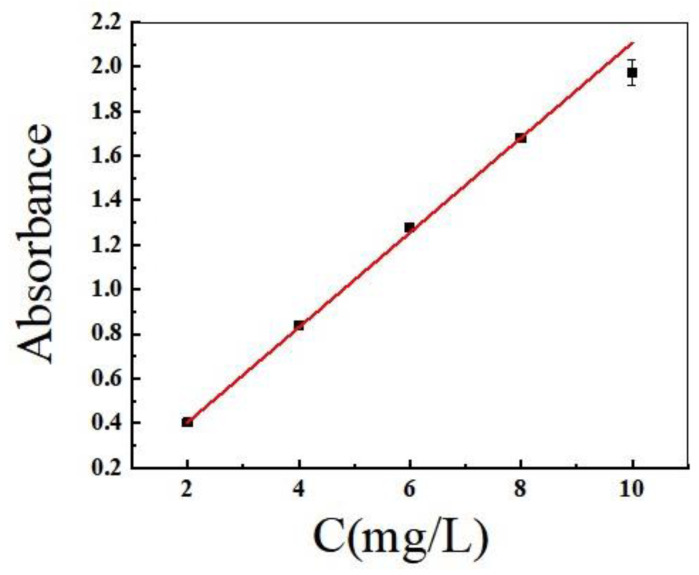
The standard curve of MB.

The analysis using spectrophotometry was conducted as follows. The stock solution was diluted at a concentration of 1000 mg/L in a 500 mL volumetric flask with ultrapure water, and the volume was set to the scale of the flask and shaken well. The concentrations were configured as 2 mg/L, 4 mg/L, 6 mg/L, 8 mg/L, and 10 mg/L, and the absorbance was measured at the maximum absorption wavelength of 665 nm. C was plotted (Figure 10), and the standard curve of MB absorbance versus its concentration was obtained as A = 0.213x − 0.02, R^2^ = 0.9996. The adsorption amount of MB by biochar was calculated according to the following equation:

(2) $$q_{e}=\frac{C_{0-}C_{e}V}{M}$$

(3) $$q_{t}=\frac{C_{0-}C_{t}V}{M}\;\;$$

where C_0_, C_e_, and C_t_ are the initial concentration of the solution, the concentration at adsorption equilibrium, and the concentration of the solution at a certain moment, mg/L, respectively; V is the volume of the adsorption solution, L; m is the mass of the adsorbent, g; q_e_ and q_t_ are the amount of adsorption at equilibrium and the amount of adsorption at a certain moment, mg/g, respectively; V(L) is the volume of the solution; and M is the amount of biochar.

(2) Formulation of methylene blue solutions in adsorption isotherms and adsorption kinetics

Adsorption experiments were carried out using methylene blue as the adsorbent to determine the adsorption performances of activated carbons. The methylene blue mother liquor of 1000 mg/L was first prepared and then diluted into methylene blue standard solutions of 50, 100, 150, 200, 250, and 300 mg/L. Then, 100 mL of methylene blue solution with a concentration of 300 mg/L was added to 0.1 g of biochar in a 200 mL beaker, and the beaker was stirred on a magnetic stirrer under neutral conditions at room temperature (25 ± 2 °C). The samples were taken at different periods of 5, 15, 30, 60, 120, 240, 480, 720, and 1440 min, and the absorbance values were measured to calculate the adsorption amount and compare the effect of adsorption time on the adsorption effect. The adsorption effect was compared with the effect of the initial mass concentration of MB solution on the adsorption effect. 

#### 4.4.2. Adsorption Kinetics

Adsorption kinetics expresses the nature (physical or chemical) of the interaction dependence between adsorbate and adsorbent and is important for evaluating the mechanism and efficiency of the adsorption process [33,34]. The kinetic constant k is used to determine the rapidity of the adsorption rate.

(1) Pseudo-first-order kinetic equation

The model equation is as follows: the adsorption rate constant is determined by the quasi-first-order equation given by Langergen and Svenska. The equation is expressed as:

(4) $$\mathrm{lg}\left(q_{e}-q_{t}\right)=\mathrm{lg}q_{e}-k_{1}t/2.303$$

where *q_e_* is the adsorption amount at equilibrium (mg/g); *q_t_* is the adsorption amount at t (mg/g); *K_2_* is the secondary rate constant (g/(mg-min^−1^); and t is the adsorption time (min).

(2) Pseudo-second-order kinetic equation

The pseudo-second-order kinetic equation can be described using the McKay equation, which indicates that the adsorption process is chemisorption controlled by van der Waals forces generated by electron transfer or sharing between the adsorbent and the adsorbate. The expression of the equation is:

(5) $$\frac{t}{q_{t}}=\frac{1}{k_{2}q_{e}^{2}}+\frac{1}{q_{e}}t$$

where *q_e_* is the adsorption amount at equilibrium (mg/g); *q_t_* is the adsorption amount at t (mg/g); *K_2_* is the secondary rate constant (g/(mg-min^−1^); and t is the adsorption time (min).

#### 4.4.3. Adsorption Isotherm Curve

Adsorption isotherms are used to assess the suitability of the adsorption process and to evaluate the maximum adsorption capacity of the adsorbent material. In addition, the fit of the isotherm model to the experimental data provides information about the adsorption properties. There are several equations for adsorption equilibrium data, among which the commonly used surface adsorption models for single solute systems are Langmuir [34] and Freundlich [35].

(1) Langmuir isotherm

The model assumes that adsorption occurs at the same locations on the surface of the same adsorbent, that these same locations have the same availability and energy equivalence, that each location carries the same number of molecules, and that there are no interactions between the adsorbed molecules. The expression is as follows:

(6) $$\frac{c_{e}}{q_{e}}-\frac{1}{bq_{m}}+\frac{c_{e}}{q_{m}}$$

where *q_e_* is equilibrium adsorption capacity (mg/g); b is the Langmuir constant related to the heat of adsorption (L/mg); C_e_ is equilibrium concentration (mg/L); and *q_m_* is maximum adsorption capacity of the adsorbent (mg/g).

(2) Freundlich isotherm model

The Freundlich adsorption isotherm assumes that adsorption occurs on uneven surfaces of the adsorbent and can be reasonably applied over a low-to-moderate range of adsorbate concentrations. The expression is as follows:

(7) $$\mathrm{lg}q_{e}=\mathrm{lg}K_{F}+1/n\mathrm{lg}c_{e}$$

where *q_e_* is equilibrium adsorption capacity (mg/g); *K_F_* is Freundlich adsorption isotherm constant; n is adsorption strength; and C_e_ is equilibrium concentration (mg/L).

## Figures and Tables

**Table 1:** Organic composition analysis, ash composition, and yield analysis of three kinds of biomass.

Samples	Organic Composition Analysis/%			Ash%	Yield	
	Lignin	Cellulose	Hemicellulose		Biomass_400°C_	BiomassCO_2_
WGH	12.76	24.65	8.55	11.30	38.80	30.33
SB	21.69	47.38	25.60	1.20	31.50	23.81
RS	20.32	39.81	15.81	7.21	35.00	24.26

**Table 2:** Statistical table of porosity and specific surface area of different samples.

Samples	S_BET_ (m^2^/g)	V_total_ (cm^3^/g)	V_mic_ (cm^3^/g)	V_mic_/V_t_ (%)	D_ap_ (nm)
WGB	6.57	0.0287	0.0058	20	7.86
SBB	24.85	0.0960	0.0207	22	8.09
RSB	15.63	0.0649	0.0143	22	5.52
WGB-CO_2_	15.53	0.0210	0.0095	45	5.64
SBB-CO_2_	352.99	0.2030	0.1698	84	2.36
RSB-CO_2_	215.0447	0.1509	0.0849	56	2.8481

**Table 3:** Content of functional groups on the surface of CO_2_ biomass before and after activation.

	C1s	Peak Position			Content%		
		C1	C2	C3	C1	C2	C3
WGB	78.10	284.68	286.58	288.48	80.65	10.48	8.87
SBB	86.31	284.8	263.38	288.28	80.00	14.4	5.60
RSB	55.93	284.7	286.58	289.28	75.76	12.12	12.12
WGB-CO_2_	79.17	284.81	286.38	293.38	69.44	19.44	11.30
SBB-CO_2_	92.78	284.78	286.08	287.08	80.65	12.6	6.45
RSB-CO_2_	76.02	284.79	286.48	293.68	63.51	21.16	15.33

**Table 4:** Adsorption capacity of biochar treated with different acids and bases.

Time/min	WGB	SBB	RSB	WGB-CO_2_	SBB-CO_2_	RSB-CO_2_
5	63.15	93.90	78.64	46.48	127.70	91.90
15	65.02	97.89	85.21	63.15	137.09	97.89
30	71.36	106.10	86.38	65.73	142.49	106.81
60	72.30	106.57	86.85	69.72	151.17	111.97
120	73.00	106.81	86.62	85.21	163.62	115.26
240	72.54	107.04	87.32	85.45	177.46	117.37
480	73.47	107.04	87.32	85.68	177.93	119.01
720	74.65	107.28	87.56	85.92	178.17	119.25

**Table 5:** Kinetic parameters of quasi-first-order and quasi-second-order adsorption equations of different original biochars.

Smaples	q_exp_ (mg/g)	Pseudo-First-Order Kinetic Model			Pseudo-Second-Order Kinetic Model		
		k_1_ (min)^−1^	q_e_ (mg/g)	R^2^	k_2_ (min)^−1^	q_e_ (mg/g)	R^2^
WGB	74.65	1.54 × 10^−3^	18.77	0.6209	−4.32 × 10^−5^	73.53	0.9997
SBB	107.28	1.31 × 10^−3^	2.96	0.5204	−1.86 × 10^−5^	107.18	0.9999
RSB	87.56	1.09 × 10^−3^	2.15	0.6015	−2.93 × 10^−5^	87.41	0.9999
WGB-CO_2_	85.92	2.07 × 10^−3^	18.63	0.7559	−3.00 × 10^−5^	87.18	0.9994
SBB-CO_2_	178.17	5.63 × 10^−3^	133.29	0.9796	−5.63 × 10^−6^	185.19	0.9995
RSB-CO_2_	119.25	2.63 × 10^−3^	16.61	0.9559	−1.47 × 10^−5^	119.62	0.9999

**Table 6:** Parameters of the adsorption isotherm model for different treatments of biochar.

Samples	q_exp_ (mg/g)	Langmuir			Freundlich		
		q_m_	K_L_	R^2^	k_F_	n	R^2^
WGB	74.41	119.19	0.01	0.8606	1.59	1.55	0.8758
SBB	104.69	121.36	0.02	0.9026	2.78	2.42	0.9607
RSB	84.51	90.99	0.05	0.9278	3.75	3.73	0.6907
WGB-CO_2_	82.39	114.68	0.01	0.9415	1.97	1.85	0.8635
SBB-CO_2_	121.24	176.06	0.39	0.9746	7.84	12.29	0.9728
RSB-CO_2_	119.95	137.93	0.03	0.9190	3.70	2.98	0.9287

## Data Availability

The data presented in this study are available on request from the corresponding author.
